# Social network characteristics and levels of fluctuations in momentary depressive symptomatology among older adults

**DOI:** 10.1136/jech-2024-222959

**Published:** 2025-08-14

**Authors:** Vernon Cail, Mariëlle A Beenackers, Frank J Van Lenthe, Joost Oude Groeniger, Giovanna Fancello, Andrea Montanari, Cédric Sueur, Yan Kestens, Julie Vallée, Basile Chaix

**Affiliations:** 1 Department of Public Health, Erasmus MC University Medical Center Rotterdam, Rotterdam, Zuid-Holland, The Netherlands; 2 INSERM, Institut Pierre Louis d’Epidémiologie et de Santé Publique, Sorbonne Université, Paris, Île-de-France, France; 3 IPHC UMR7178, CNRS, Université de Strasbourg, Strasbourg, Alsace, France; 4 ANTHROPO-LAB, ETHICS EA 7446, Université Catholique de Lille, Lille, Hauts-de-France, France; 5 Institut Universitaire de France, Paris, Île-de-France, France; 6 École de santé publique, Université de Montréal, Montreal, Quebec, Canada; 7 Centre de recherche en santé publique, Université de Montréal, Montreal, Quebec, Canada; 8 UMR Géographie-cités, CNRS, Paris-Aubervilliers, Île-de-France, France; 9 UMR LISST, CNRS, Toulouse, Île-de-France, France

**Keywords:** DEPRESSION, PUBLIC HEALTH, Healthy Aging

## Abstract

**Background:**

Social networks are known to protect against depressive symptoms in older adults. However, most research relies on retrospective self-reported depression measures and cross-sectional data, which may introduce bias. Ecological momentary assessment with longitudinal data overcomes these limitations by repeatedly measuring the subject’s experience in the present moment. This study examined how social network characteristics relate to momentary depressive symptoms and their daily fluctuations in older adults.

**Methods:**

We analysed data from 216 older adults in Paris, France, using the Healthy Aging and Networks in Cities and Promoting Mental Well-Being and Healthy Aging in Cities studies. Social network characteristics included network size and frequency of in-person and digital interactions per week. Depressive symptomatology was assessed using a daily smartphone survey of the Center for Epidemiological Studies-Depression over a week. Linear mixed-effect models estimated associations between social network characteristics and momentary depressive symptoms, while multivariable linear models examined relationships with daily symptom fluctuations.

**Results:**

Network size and frequency of contact from digital communications per week were not associated with fewer depressive symptoms; however, there was suggestion that having more in-person contact was related to fewer depressive symptoms (exp(β) = 0.90, 95% CI 0.82 to 1.00). Moreover, having a larger social network (exp(β) = 0.91, 95% CI 0.85 to 0.98) and more in-person contacts (exp(β) = 0.96, 95% CI 0.93 to 0.98) were associated with less fluctuations in daily depressive symptoms, but no association for the frequency of contact from digital communications was observed.

**Conclusion:**

Findings from this study suggest that larger social networks and more in-person contact may promote more stable and better mental health among older adults.

**WHAT IS ALREADY KNOWN ON THIS TOPIC**

- Depression in older adults is an important public health issue, especially since the world’s population of people aged 60 years and older is expected to rise rapidly. Previous research has shown that certain social network characteristics protect against depressive symptoms in older adults. However, these findings are largely based on retrospective self-reported measures of depression and cross-sectional analyses, which are prone to measurement error, recall bias and reverse causation.

**WHAT THIS STUDY ADDS**

- Ecological momentary assessment uses repeated collection of real-time data on subjects’ behaviour and experience in their natural environments. Using a longitudinal dataset with a baseline survey and repeated measurements of depression, this study found that having more frequent in-person contacts with members of the social network was related to less depressive symptoms and that having a larger social network and more frequent in-person contacts with members of the social network were associated with less fluctuations in depressive symptoms.

**HOW THIS STUDY MIGHT AFFECT RESEARCH, PRACTICE OR POLICY**

- This study provides further evidence that larger social networks and more in-person interactions might mitigate daily fluctuations in depressive symptoms among older adults. Developing interventions (eg, in the form of contexts or events) that provide opportunities for building social ties could be a viable approach to promote a stable mental health among older adults.

## Introduction

 Depression in older adults is an important public health issue. Older adults with depression have a higher risk of morbidity, suicide and mortality from other causes as well as less physical, cognitive and social functioning compared with non-depressed older adults.^1^ A recent systematic review and meta-analysis found that the overall global prevalence of depressive symptoms in older adults was 28.4%.^2^ Further complicating matters, it is estimated that one in six people in the world will be aged 60 years or over by 2030, and this number is expected to increase to 2 billion people by 2050.^3^ Accordingly, this rapid growth in the world’s population of people aged 60 years and older presents significant challenges to public health officials to prevent suffering and reduce healthcare costs related to depression in older adults.

Social relationships could play a crucial role in preventing and mitigating depression among older adults.^4^ The social network, for example, is considered a key determinant of healthy ageing.^5^ Social networks refer to the structure of social relationships and consist of multiple characteristics, such as the size of the network and the frequency of interactions.^5^ Considered a structural basis for social capital within public health research,^6^ social networks provide access to material resources and various forms of support and can buffer the stress of adverse life events.^5^ Such benefits are important to older adults who face declining mobility and more social isolation. Understanding the impact of social networks on depressive symptomatology in older adults is important for developing effective gerontological interventions.

Research on the influence of social network characteristics on depression has gained much needed attention. However, the evidence for certain social network characteristics is inconclusive and this research is marred by limitations.^7^ A 2015 systematic review found that a larger social network was a protective factor against depressive symptoms, but it did not find consistent evidence for the role of frequency of social contact on depression.^7^ The majority of the studies relied on retrospective self-reports to assess depressive symptomology, which is subject to recall distortion and can lead to measurement errors.^8^ Furthermore, the analysis of purely cross-sectional survey data amplifies the risk of reverse causation, as depression can lead people to withdraw from their social network.

One approach that can attenuate recall error and memory distortion is ecological momentary assessment (EMA).^8^,^10^ EMA refers to a range of methods that uses repeated collection of real-time data on subjects’ behaviour and experience in their natural environments.^11^ The appeal in applying EMA methodologies lies in its ability to assess the state of the participants’ symptoms as they are experiencing it. As a result, EMA measurements, especially when repeated assessments are conducted for each participant, are considered more accurate than one-time, retrospective measurements.^8 10^ Furthermore, if measurements of the exposure (ie, social networks structure) precede EMA measurements, then reverse causation can be minimised.

Another important application of EMA lies in its ability to detect fluctuations of symptoms over time.^10^ Depressive symptoms are often characterised as dynamic processes that fluctuate in response to external environmental demands and internal regulatory forces.^12^ Examining such fluctuations, also referred to as affective dynamics, offers further insight into psychological functioning and well-being. A widely used index of affective dynamics is the within-person variability calculated with the standard deviation (SD).^13^ This index captures the overall range of affect levels an individual may experience across a particular time period, where high variability indicates larger deviations from average emotional levels.^13^ In general, higher variability in negative affect has been shown to be associated with adverse mental health and well-being such as lower levels of psychological well-being^14^ and higher risk for mild cognitive impairment.^15^ However, to date, no study has examined the relationship between social network characteristics and fluctuations in depressive symptoms.

The first aim of this analysis is to assess the relationship between social network characteristics and momentary depressive symptomatology in older adults. We hypothesise that a larger social network and a higher frequency of contacts with members of the social network will be associated with fewer depressive symptoms. The second aim is to assess the relationship between social network characteristics and fluctuations in daily depressive symptoms. We hypothesise that a larger social network and a higher frequency of contacts will be associated with less variability in daily depressive symptoms.

## Methods

### Study design

The present analysis was based on a sample of 216 older adults (aged 60 and over) from the Paris region (France), which was collected from July 2019 to July 2021.^16^ The Healthy Aging and Networks in Cities (HANC) and Promoting Mental Well-Being and Healthy Aging in Cities (MINDMAP) sensor studies recruited participants among the 5500 participants of the second wave of the RECORD study.^16^,^18^ Inclusion criteria were being aged 60 or older, able to provide informed consent and capable of completing a 7-day EMA protocol via smartphone, whereas exclusion criteria included severe cognitive impairment or inability to use the mobile application.^17^ We have shown that the RECORD study itself oversampled males, educated people and residents of high socioeconomic status neighbourhoods and low building density neighbourhoods.^19^ The selection of HANC and MINDAP participants within RECORD added another layer of selection; therefore, although the present study included a diversity of participants, it is not representative of the background population of the Paris region.

Using the framework of the HANC and MINDMAP projects, this analysis obtained information on participants’ socioeconomic profiles, their social network characteristics and their momentary depressive symptoms.^17 18 20^ Momentary depressive symptoms were measured using smartphone-based EMA application over a 7-day period.^17^ Participants were asked about their current mood four times a day at random times within the following slots: 9:00 a.m.–12:00 a.m., 12:00 a.m.–2:00 p.m., 2:00 p.m.–4:00 p.m. and 4:00 p.m.–6:00 pm.^17^ Prior to the EMA data collection, information about the respondents’ sociodemographic profile was collected with a computer assisted personal interview questionnaire, and information about the respondents’ social network was collected with VERITAS, an interactive map-based questionnaire.^21^ Since data collection coincided with the COVID-19 pandemic, participants who were surveyed after the lockdown (17 March 2020–28 May 2021) were asked to answer questions regarding their social interactions and their social network before the lockdown. In-person briefings and ongoing technical support were provided to optimise compliance to the protocol.

### Dependent variable

Depressive symptoms were assessed each day in the 7-day period with the short eight-item version of the Center for Epidemiological Studies-Depression (CES-D8) scale, a widely used and validated instrument of depression in large scale surveys.^22^ Participants were asked two of the eight items within the four aforementioned time slots so that all eight items from the CES-D8 were offered during each day. The order of the items differed across days and participants, and participants were required to submit their answers within 90 min. The original items were translated to French and rephrased to inquire about the present moment as the following: ‘At the moment, I…’ (1) ‘feel bothered by things that usually do not bother me’, (2) ‘feel depressed’, (3) ‘feel that everything done is an effort’, (4) ‘feel happy’, (5) ‘feel lonely’, (6) ‘enjoy life’, (7) ‘feel sad’ and (8) ‘feel unmotivated or uninspired’. The four response options included ‘no, not at all’, ‘no, not really’, ‘yes, somewhat’ and ‘yes, absolutely’ and ranged from zero to three, respectively. The two positive items were reverse coded to reflect that a higher score indicates more depressive symptoms. A total score was calculated for each participant per day by summing the items. The daily CES-D8 score could range from 0 to 24. Some participants did not respond in the appropriate timeframe, which resulted in missing values. A summary of missingness can be found in online supplemental table 1. Within-person fluctuations in depressive symptoms were measured as the SD of each participant’s daily CES-D8 scores. The SD was calculated for each participant based on their CES-D8 scores across the week.

### Independent variable

Prior to the EMA ambulatory study, respondents were asked to describe each member of their social network and how they are connected, yielding a number of social network characteristics.^20 23^ The size of the network and the frequency of contacts of the social network served as independent variables. Network size was defined as the number of people with whom the respondent usually performed social activities. Respondents were also asked how often they usually have contact with the members of their social network in a week from two modes of communication: digital communications such as internet and phone calls and in-person communication. Each social network variable was scaled to make the regression coefficients more understandable. The network size was divided by ten. Hence, a one unit increase in the scaled network size signifies an increase in 10 persons in the network size. Both frequency of contacts variables (digital and in-person) were divided by 50, which means a one unit increase represents an increase in 50 interactions in a week.

### Covariates

The following variables were taken into account to neutralise potential confounding effects: age (in years), gender (male; female), marital status (married/in a couple; divorced/widowed/unmarried), monthly household income per consumption unit (<€2000; €2000–4000; >€4000), employment status (employed; retired/other) and general health status self-assessed before the ambulatory study (good health; poor health). Following the work from Fancello and colleagues, education level was categorised as low-medium education (from none to the completion of high school), high education (high school +2 to 4 years) and very high education (high school +5 years and over).^24^ A binary variable was incorporated to account for the effects of the pre- and post-lockdown periods.

### Data analysis

Descriptive analyses were conducted to characterise the study population, social network indicators and depressive symptoms. The first part of the main analysis estimated the association between the social network characteristics and momentary depressive symptoms with three separate mixed effects linear models, one for each social network characteristic. Mixed effects models have the ability to take into account the hierarchical structure of nested data.^25^ In this analysis, repeated daily measurements of CES-D8 (ie, level-1) were nested within participants (ie, level-2). In the second main analysis, three separate multivariable linear models were employed to estimate the association between the social network characteristics and the SD of the daily CES-D8 scores. The CES-D8 scores and the SD of the daily CES-D8 scores had skewed distributions. Therefore, we applied a natural logarithm transformation on both dependent variables. Because there were zero values, we used a log(x+1) transformation to prevent undefined values at zero. All coefficients and their respective confidence intervals (CI) were exponentiated, and exponentiated coefficients of log-linear models can be interpreted as multipliers of the outcome of interest. As part of the sensitivity analysis, we reran both models with non-transformed outcomes. All models were adjusted for gender, age, marital status, education, household income, pre-ambulatory study health status and COVID-19 pandemic period.

Missing variables, including missing depressive symptoms reports, were handled by using the multivariate imputation by chained equations package in R.^26^ Twenty imputed datasets were generated and pooled together to report the estimates and their 95% CI. Following best practices for EMA studies, we tested model assumptions and verified consistency across both complete-case and imputed datasets (not reported). All analyses were conducted in R 4.2.2.^27^

## Results

### Descriptive analysis

Table 1 provides an overview of the study population demographics. Among the 216 older adults, 64% (n=139) were males, 67% (n=145) were married or in a couple and 84% (n=183) were retired or not employed. The average age of the study sample was 70.1 years (SD=6.1). On average, participants completed 6.8 full CES-D8 surveys (SD=0.4). The average size of the social network was 6.5 (SD=3.2; IQR 4,8). Participants had, on average, 65.3 in-person contacts per week (SD=44.6; IQR 33.5, 84.5) and 106.7 digital communications (SD=76.5, IQR 59, 142.5) per week.

**Table 1:** Characteristics of the study population

	N (%) or mean (SD)	Range (min, max)
Sample size, n (%)	216 (100)	
*Individual characteristics*		
Age, mean (SD)	70.1 (6.1)	(60, 90)
Gender, n (%)		
Female	77 (35.6)	–
Male	139 (64.4)	–
Marital status, n (%)		
Married/in a couple	145 (67.1)	–
Divorced/widowed/unmarried	71 (32.9)	–
Education level, n (%)		
Low-medium	65 (30.1)	–
High education	70 (32.4)	–
Very high education	81 (37.5)	–
Household income, n (%)		
<2000	32 (14.9)	–
2000–4000	128 (59.2)	–
>4000	56 (25.9)	–
Employment, n (%)		
Employed	29 (13.4)	–
Retired/unemployed/other	183 (84.7)	–
Other	4 (1.9)	–
Self-assessed health, n (%)		
Good health	198 (91.7)	–
Poor health	17 (7.8)	–
NA	1 (0.5)	
COVID-19 lockdown, n (%)		
Pre-lockdown	76 (35.1)	–
Post-lockdown	139 (64.3)	–
NA	1 (0.5)	–
Social network characteristics, mean (SD)		
Network size	6.5 (3.2)	(1, 19)
In-person contacts per week	64.8 (44.5)	(0, 247)
Digital communications per week	42.5 (54.4)	(0, 544)
Number of completed CES-D8 per participant, mean (SD)	6.8 (0.4)	(5, 7)
CES-D8 scores, mean (SD)*	3.6 (2.9)	(0, 21)
Within-person variability CES-D8 scores, mean (SD)*	2.2 (1.0)	(0, 9.8)

* based on imputed data.

CES-D8, Center for Epidemiological Studies-Depression.

### Association of social network characteristics with momentary depressive symptoms

Table 2 presents the associations between the social network characteristics and the log-transformed momentary depressive symptoms. For every 10 additional persons in the social network, momentary depressive symptoms were 10% lower, but the CI included 1 (exp(β) = 0.90, 95% CI 0.70 to 1.17). Regarding frequency of in-person contacts, having 50 more in-person contacts was associated with 10% less depressive symptoms. Although the CI also included 1 (exp(β) = 0.90, 95% CI 0.82 to 1.00), there was a suggestion of association. Every 50 extra digital communications was associated with 2% less depressive symptoms, but the CI overlapped value 1 (exp(β) = 0.98, 95% CI 0.91 to 1.06). All models met model assumptions and verified consistency across both complete-case and imputed datasets. The results from the sensitivity analysis were aligned with the main analysis, as there was also a suggestion of association where more in-person contacts were associated with less depressive symptoms but with a relatively wide confidence interval (online supplemental table 2).

**Table 2:** Exponentiated beta coefficients and 95% CIs from the multilevel models estimating the associations between social network characteristics and the log-transformed momentary depressive symptoms

	Network size	In-person contacts	Digital communications
Intercept	2.78 (1.06, 7.27)	3.25 (1.23, 8.58)	2.78 (1.06, 7.30)
Network size *	0.90 (0.70, 1.17)		
In-person contacts †		0.90 (0.82, 1.00)	
Digital communications †			0.98 (0.91, 1.06)
Age	1.01 (0.99, 1.02)	1.01 (0.99, 1.02)	0.01 (-0.01, 0.02)
Gender			
Female	Ref	Ref	Ref
Male	1.01 (0.99, 1.02)	1.01 (0.99, 1.02)	1.01 (0.99, 1.02)
Marital status			
Married/in a couple	Ref	Ref	Ref
Divorced/widowed/unmarried	1.07 (0.89, 1.3)	0.98 (0.79, 1.21)	1.08 (0.90, 1.31)
Education level			
Low-medium	Ref	Ref	Ref
High education	0.97 (0.79, 1.19)	0.95 (0.78, 1.17)	0.95 (0.78, 1.17)
Very high education	1.01 (0.81, 1.26)	0.99 (0.79, 1.23)	1.01 (0.81, 1.25)
Household income			
<2000	Ref	Ref	Ref
2000–4000	1.01 (0.79, 1.28)	0.98 (0.77, 1.25)	1.01 (0.79, 1.28)
>4000	1.00 (0.75, 1.33)	0.95 (0.71, 1.26)	0.99 (0.74, 1.32)
Employment			
Employed	Ref	Ref	Ref
Retired	0.86 (0.66, 1.12)	0.83 (0.64, 1.07)	0.87 (0.67, 1.13)
Unemployed/other	0.56 (0.30, 1.05)	0.56 (0.30, 1.04)	0.56 (0.30, 1.06)
Self-assessed health			
Good health	Ref	Ref	Ref
Poor health	1.28 (0.97, 1.70)	1.31 (0.99, 1.73)	1.28 (0.96, 1.70)
COVID lockdown			
Pre-lockdown	Ref	Ref	Ref
Post-lockdown	0.98 (0.82, 1.16)	0.96 (0.82, 1.13)	0.97 (0.82, 1.14)
Individual-level variance	0.301	0.296	0.302
Repeated measure variance	0.221	0.221	0.221
ICC ‡	0.576	0.572	0.577

* One unit increase means an increase by 10.

† One unit increase means an increase by 50.

‡ Intraclass correlation coefficient (ICC)is the variance between individuals divided by the total variance.

### Association of social network characteristics with fluctuation in depressive symptoms

The associations of social network characteristics with fluctuation in daily depressive symptoms, expressed as the log-transformed SD, are presented in table 3. Having 10 more members in one’s social network was associated with having 9% less daily fluctuations in depressive symptoms (95% CI 0.85 to 0.98) after controlling for sociodemographic factors. In addition, having 50 more in-person contacts in a week was associated with less variability in daily depressive symptoms by 4% (95% CI 0.93 to 0.98); however, no association was observed regarding frequency of digital contacts in a week (exp(β) = 0.99 95% CI 0.97 to 1.01). The results of the sensitivity analysis (online supplemental table3), which examined the associations between the social network characteristics and the non-transformed outcome, demonstrate that only network size (β=−0.30, 95% CI −0.48 to –0.11) and the frequency of in-person contacts (β=−0.09, 95% CI −0.16 to –0.01) were associated with a lower variability in daily depressive symptoms but not contact from digital communications (β=−0.01, 95% CI −0.06 to 0.04).

**Table 3:** Exponentiated beta coefficients and 95% CIs from the linear models estimating the associations between social network characteristics and the log-transformed SD in daily CES-D8 scores

	Network size	In-person contacts	Digital communications
Intercept	1.74 (1.34, 2.26)	1.86 (1.43, 2.42)	1.74 (1.34, 2.26)
Network size *	0.91 (0.85, 0.98)		
In-person contacts †		0.96 (0.93, 0.98)	
Digital communications †			0.99 (0.97, 1.01)
Age	1.01 (1.00, 1.01)	1.01 (1.00, 1.01)	1.01 (1.00, 1.01)
Gender			
Female	Ref	Ref	Ref
Male	1.02 (0.97, 1.07)	1.03 (0.98, 1.08)	1.03 (0.98, 1.09)
Marital status			
Married/in a couple	Ref	Ref	Ref
Divorced/widowed/unmarried	1.17 (1.11, 1.24)	1.13 (1.07, 1.20)	1.18 (1.12, 1.25)
Education level			
Low-medium	Ref	Ref	Ref
High education	0.94 (0.87, 1.02)	0.91 (0.85, 0.98)	0.92 (0.85, 0.99)
Very high education	1.02 (0.94, 1.12)	1.00 (0.92, 1.10)	1.02 (0.93, 1.11)
Household income			
<2000	Ref	Ref	Ref
2000–4000	0.84 (0.79, 0.90)	0.83 (0.78, 0.88)	0.84 (0.79, 0.89)
>4000	0.90 (0.83, 0.97)	0.87 (0.81, 0.94)	0.89 (0.82, 0.96)
Employment			
Employed	Ref	Ref	Ref
Retired	0.96 (0.89, 1.03)	0.94 (0.88, 1.01)	0.96 (0.89, 1.03)
Unemployed/other	0.77 (0.64, 0.93)	0.77 (0.64, 0.92)	0.77 (0.64, 0.93)
Self-assessed health			
Good health	Ref	Ref	Ref
Poor health	1.26 (1.16, 1.37)	1.27 (1.17, 1.38)	1.26 (1.16, 1.37)
COVID-19 lockdown			
Pre-lockdown	Ref	Ref	Ref
Post-lockdown	1.05 (1.00, 1.10)	1.04 (0.99, 1.09)	1.04 (1.00, 1.09)

* one unit increase means an increase by 10.

† one unit increase means an increase by 50.

CES-D8, Center for Epidemiological Studies-Depression.

## Discussion

This study did not find major evidence to support our hypothesis that the size of the network and the frequency of contact from digital communications per week were associated with fewer depressive symptoms. Nonetheless, although the 95% interval included value 1 just on its upper side, there was a suggestion that the frequency of in-person contact was associated with slightly fewer depressive symptoms. When examining instability in depressive symptoms, our findings indicate that the size of the social network and the frequency of in-person contacts were associated with less variability in daily depressive symptoms.

The findings on the relationship between frequency of contacts and depressive symptoms are mixed.^7^ While some studies found that more contact with the social network is associated with fewer depressive symptoms,^28 29^ others did not find any associations.^30 31^ Researchers who did not observe any association postulated that the subjective components of social relationships, like the perceptions of the quality of social relationships, might be more critical to health than objective characteristics of one’s social network.^30 31^ Our findings might provide some support for this argument. Although the CI included value 1 on the margin, we found that more in-person contacts were linked with fewer symptoms of depression, and more so than contacts with digital communication. One possible explanation is that in-person communication offers a richer form of communication than digital communication among older adults.^32^ In-person communications provide more interaction cues such as gestures, facial expressions, intonation and body language than digital communications.^32^ The lack of such cues when communicating with digital technologies could diminish older adults’ ability to convey and experience interpersonal impressions and warmth as social presence theory posits.^33^ Older adults also might lack the technical skills to properly use digital communications.^34^ Digital communications could also lead to more exposure to negative content and misinformation that proliferated on the internet during the pandemic.^35^ These barriers could suggest that digital communications are not a substitute for in-person interactions among older adults. Prior research has shown that older adults preferred in-person communication over digital communication during the COVID-19 pandemic.^36 37^

Our study adds to the literature by examining the relationship between social network characteristics and fluctuations in depressive symptoms. Our findings confirm our hypothesis that older adults with larger social networks and more in-person contact with their social network had less variability in depressive symptoms across a week. These results are in line with prior cross-sectional research emphasising the protective effects of network size on depression in older adults.^7 38^ By relying on repeated real-time measures, our EMA-based design strengthens the validity of the associations between social networks and depressive symptoms in older adults by minimising recall bias and allowing us to capture fluctuations in symptomatology, which are often missed in traditional designs.

Our findings provide evidence that social networks might buffer against the adverse effect of stressful events.^39^ During the COVID-19 pandemic, many people experienced elevated and sporadic stress levels, especially older adults who were among the high-risk groups for adverse health effects from the COVID-19 virus. We believe participants’ social networks may have mitigated sudden spikes in stress through social support. Additionally, participants with large networks and more in-person interactions may be less likely to feel loneliness, which could contribute to fluctuations in depressive symptoms.

### Strengths and limitations

This study goes beyond existing literature by taking an EMA approach to assess momentary depressive symptoms, which is the main strength of this study. Moreover, social network characteristics were assessed prior to the assessment of depressive symptomatology. Although this approach does not eliminate reverse causation, we believe it minimises it. There are some limitations that should also be considered. First, social network characteristics were assessed using a self-reported, time-invariant measure, which is susceptible to recall and reporting biases. Moreover, our sample was not representative of older adults in Paris due to selective participation bias,^19^ which makes it difficult to generalise the results to the broader population. In addition, nearly two-thirds of our sample has been surveyed after the lockdown and was asked to answer questions about their social network before the lockdown. The threat of reverse causation remains, if an underlying depression status led to a reduction in the social network or in the frequencies of contacts. However, this is not an easy issue to solve, as we believe that adjusting for a CES-D8 indicator that was collected at the same time as the characterisation of the social network may have represented an overadjustment of the model. Finally, although SD is widely used for measuring the variability of affect levels, it does not take into account how affect changes over time.^40^ The mean square successive difference is considered the preferred index of affective instability, because it captures both variability and temporal dependency.^40^

## Conclusions

The findings from this study suggest that certain social network characteristics protect against depressive symptoms in terms of levels but also stability. Given the rapid growth in the world’s elderly population and the burden associated with depression, our findings have important policy implications. Intervention programmes that provide older adults the opportunity to increase in-person interactions and to increase the size of their social networks such as providing contexts or events to meet are recommended to promote mental health.

## Supplementary material

10.1136/jech-2024-222959 online supplemental file 1

## Data Availability

Data are available upon reasonable request.
