# Supplementary material for: Social network characteristics and levels of fluctuations in momentary depressive symptomatology among older adults
Source: J Epidemiol Community Health. 2025 Aug 14;79(11):e222959. doi: 10.1136/jech-2024-222959 (PMC12573400; doi:10.1136/jech-2024-222959)
Supplement: online supplemental file 1 [file jech-79-11-s001.pdf]

Appendix

Supplement Table 1. Description of missing CES-D8 items

Average number of missing EMA responses per respondent across the ambulatory period	7.2
Missing values by items n, (%)	
Item 1	200 (12.7)
Item 2	187 (11.8)
Item 3	220 (13.9)
Item 4	198 (12.5)
Item 5	173 (10.9)
Item 6	212 (13.4)
Item 7	196 (12.4)
Item 8	188 (11.9)
Total	1,574
Number of person-days with missing values n, (%)	
Person-days with no missing values	882 (62.8)
Person-days with one missing value	97 (6.9)
Person-days with two missing values	154 (10.9)
Person-days with three missing values	103 (7.3)
Person-days with four missing values	53 (3.7)
Person-days with five missing values	59 (4.2)
Person-days with six missing values	39 (2.7)
Person-days with seven missing values	17 (1.2)
Total number of person-days	1,404

Supplement Table 2. Beta coefficients and 95% confidence intervals from the multilevel models estimating the associations between social network characteristics and momentary depressive symptoms

	Network size	In-person contacts	Digital communications
Intercept	2.23 (-1.71, 6.17)	2.84 (-1.15, 6.82)	2.23 (-1.73, 6.19)
Network size [a]	-0.59 (-1.62, 0.44)		
In-person contacts [b]		-0.40 (-0.81, 0.01)	
Digital communications [b]			-0.09 (-0.39, 0.22)
Age	0.03 (-0.03, 0.09)	0.03 (-0.03, 0.09)	0.03 (-0.03, 0.09)
Gender			
Female	Ref	Ref	Ref
Male	-0.40 (-1.16, 0.35)	-0.34 (-1.08, 0.39)	-0.33 (-1.08, 0.41)
Marital status			
Married/ In a couple	Ref	Ref	Ref
Divorced/widowed/unmarried	0.38 (-0.41, 1.17)	0.02 (-0.86, 0.9)	0.43 (-0.35, 1.22)
Education level			
Low-medium	Ref	Ref	Ref
High education	-0.23 (-1.08, 0.61)	-0.30 (-1.13, 0.53)	-0.30 (-1.14, 0.54)
Very high education	-0.02 (-0.92, 0.88)	-0.10 (-1.00, 0.79)	-0.03 (-0.94, 0.87)
Household income			
<2,000	Ref	Ref	Ref
2,000 – 4,000	0.07 (-0.91, 1.05)	-0.05 (-1.03, 0.93)	0.05 (-0.93, 1.04)
>4,000	0.28 (-0.89, 1.45)	0.05 (-1.12, 1.22)	0.21 (-0.96, 1.38)
Employment			
Employed	Ref	Ref	Ref
Retired	-0.45 (-1.51, 0.62)	-0.60 (-1.68, 0.47)	-0.39 (-1.47, 0.68)
Unemployed/other	-1.96 (-4.53, 0.62)	-1.98 (-4.55, 0.58)	-1.92 (-4.52, 0.67)
Self-assessed health			
Good health	Ref	Ref	Ref
Poor health	1.21 (0.04, 2.38)	1.28 (0.11, 2.45)	1.21 (0.03, 2.38)
COVID lockdown			
Pre-lockdown	Ref	Ref	Ref
Post-lockdown	0.07 (-0.61, 0.76)	-0.02 (-0.69, 0.65)	0.02 (-0.66, 0.70)
Individual-level variance	5.045	4.992	5.081
Repeated measure variance	3.667	3.666	3.666
ICC [c]	0.579	0.577	0.581

[a] one unit increase means an increase by 10, [b] one unit increase means an increase by 50, [c] intra-level correlation coefficient

Supplement Table 3. Beta coefficients and 95% confidence intervals from the linear models estimating the associations between social network characteristics and standard deviation in CES-D8 scores

	Network size	In-person contacts	Digital communications
Intercept	0.97 (0.28, 1.67)	1.10 (0.39, 1.80)	0.96 (0.26, 1.66)
Network size [a]	-0.30 (-0.48, -0.11)		
In-person contacts [b]		-0.09 (-0.16, -0.01)	
Digital communications [b]			-0.01 (-0.06, 0.04)
Age	0.01 (0.00, 0.02)	0.01 (0.00, 0.02)	0.01 (0.00, 0.02)
Gender			
Female	Ref	Ref	Ref
Male	-0.01 (-0.15, 0.12)	0.03 (-0.1, 0.16)	0.03 (-0.1, 0.16)
Marital status			
Married/ In a couple	Ref	Ref	Ref
Divorced/widowed/unmarried	0.37 (0.23, 0.51)	0.31 (0.15, 0.46)	0.39 (0.25, 0.54)
Education level			
Low-medium	Ref	Ref	Ref
High education	-0.08 (-0.22, 0.07)	-0.11 (-0.26, 0.03)	-0.11 (-0.26, 0.03)
Very high education	0.04 (-0.13, 0.20)	0.01 (-0.16, 0.17)	0.02 (-0.15, 0.19)
Household income			
<2,000	Ref	Ref	Ref
2,000 – 4,000	-0.56 (-0.73, -0.39)	-0.59 (-0.76, -0.42)	-0.57 (-0.74, -0.40)
>4,000	-0.29 (-0.50, -0.09)	-0.36 (-0.57, -0.15)	-0.33 (-0.53, -0.12)
Employment			
Employed	Ref	Ref	Ref
Retired	0.03 (-0.16, 0.23)	0.01 (-0.18, 0.20)	0.05 (-0.14, 0.24)
Unemployed/other	-0.51 (-1.01, 0.00)	-0.52 (-1.02, -0.02)	-0.52 (-1.02, -0.01)
Self-assessed health			
Good health	Ref	Ref	Ref
Poor health	0.87 (0.64, 1.09)	0.87 (0.65, 1.10)	0.85 (0.63, 1.08)
COVID-19 lockdown			
Pre-lockdown	Ref	Ref	Ref
Post-lockdown	0.05 (-0.07, 0.18)	0.01 (-0.11, 0.13)	0.02 (-0.11, 0.14)

[a] one unit increase means an increase by 10, [b] one unit increase means an increase by 50
